# Utilizing Human-centered Design and Innovation to Mitigate Adverse Drug Events

**DOI:** 10.1097/pq9.0000000000000833

**Published:** 2025-08-29

**Authors:** Jenna Merandi, Erin Ahrens

**Affiliations:** From the * Pharmacy Department, Nationwide Children’s Hospital, Columbus, Ohio; † Information Services Department, Nationwide Children’s Hospital, Columbus, Ohio.

## Introduction:

The increasing complexity of healthcare delivery requires innovation to improve patient and employee safety. Traditional safety programs must evolve to engage frontline clinicians as active participants in identifying system vulnerabilities and designing solutions to mitigate errors. The roller clamp, a commonly used intravenous (IV) line clamp, is a core component of medication and fluid delivery. For more than 90 years, the simplistic, gravity-based design has remained unchanged, despite major advancements in smart infusion pump technology. If a life-sustaining medication is inadvertently clamped while a medication is being delivered, it may lead to the medication not reaching the patient, which could lead to potential harm or even a fatality if left unnoticed.

## Methods:

A review was conducted at Nationwide Children’s Hospital evaluating more than 40 safety events involving clamped lines over a 2-year period. Initial mitigation strategies focused on improving line management practices, reinforcing double-check procedures, and providing targeted staff education. However, a common cause analysis revealed deeper system- and human-based contributors to these events, highlighting the limitations of procedural interventions alone.

In response, a human-centered design initiative was launched in collaboration with Priority Designs, supported by $85,000 in funding from the hospital’s Office of Technology and Commercialization. The 4-phase project included context immersion, concept development, functional prototyping, and comparative usability testing.

Human factors engineers partnered with team members to observe frontline staff interacting with IV roller clamps in patient care environments. These immersive observations provided critical insight into how clamps are used in real-world scenarios, revealing subtle usability challenges that contribute to error. These insights directly informed the concept development phase. Of the 5 initial design concepts generated, 3 were selected for prototyping based on their potential to reduce harm. All concepts emphasized clear, intuitive visual indicators of clamp status, whereas each offered distinct safety-enhancing features. Usability testing of the design concepts was conducted with frontline clinicians. Clinical scenarios were simulated with prototypes of each concept for evaluation by frontline clinicians. Objective scoring criteria for design function were collected along with feedback on elements that were most impactful for safe and effective use.

## Results:

**Fig. 1.**
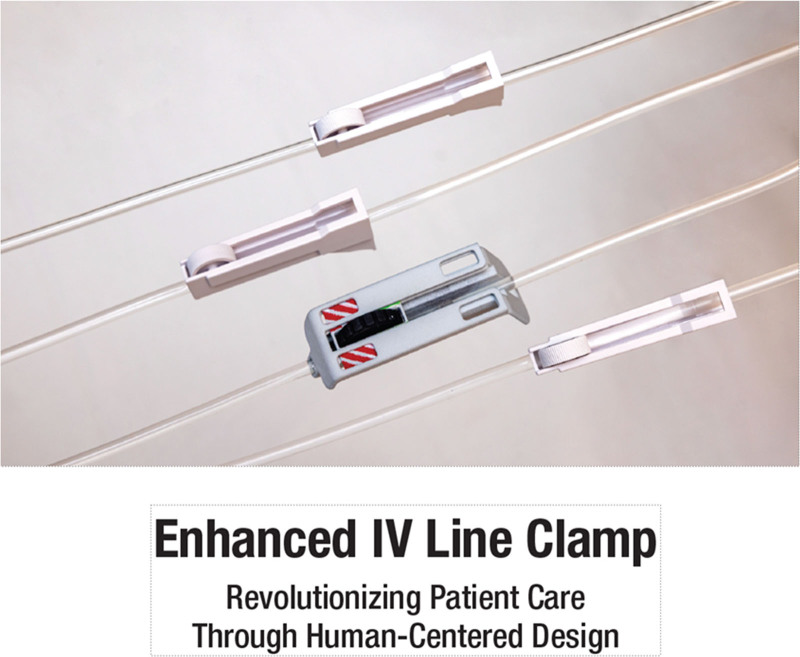
Enhanced IV-line clamp concept, compared with the commonly available roller clamp.

Analysis of usability testing identified 1 concept as the most favored, while offering opportunities for additional design considerations. Further enhancements to the concept included visual indicators to ensure accessibility for individuals with color blindness, clear, unmistakable wheel placement indicating clamp status, and enhanced visibility from multiple angles and distances (Fig. 1). Patents for the enhanced IV-line clamp designed from this project have been filed as the project team seeks to move this product toward commercialization.

## Conclusions:

This initiative demonstrates the power of human-centered design and collaboration in addressing persistent safety risks in health care. By embedding frontline clinicians in the design and testing process and applying human factors engineering within simulated clinical environments, the project produced anenhanced IV-line clamp that is better aligned with real-world clinical needs. The resulting design enhances visibility and usability driven by end-user insight to reduce preventable harm. This work represents a meaningful step toward advancing patient safety through practical device redesign.

